# Multiparameter Phospho-Flow Analysis of Lymphocytes in Early Rheumatoid Arthritis: Implications for Diagnosis and Monitoring Drug Therapy

**DOI:** 10.1371/journal.pone.0006703

**Published:** 2009-08-20

**Authors:** Carole L. Galligan, Janet C. Siebert, Katherine A. Siminovitch, Edward C. Keystone, Vivian Bykerk, Omar D. Perez, Eleanor N. Fish

**Affiliations:** 1 Toronto General Research Institute, University Health Network, Toronto, Ontario, Canada; 2 CytoAnalytics, Analytical Services, Denver, Colorado, United States of America; 3 Mount Sinai Hospital Samuel Lunenfeld and Toronto Hospital Research Institutes, Toronto, Ontario, Canada; 4 University of Toronto and Mount Sinai Hospital, Toronto, Ontario, Canada; 5 The Baxter Laboratory for Genetic Pharmacology, Stanford University School of Medicine, Stanford, California, United States of America; New York University School of Medicine, United States of America

## Abstract

**Background:**

The precise mechanisms involved in the initiation and progression of rheumatoid arthritis (RA) are not known. Early stages of RA often have non-specific symptoms, delaying diagnosis and therapy. Additionally, there are currently no established means to predict clinical responsiveness to therapy. Immune cell activation is a critical component therefore we examined the cellular activation of peripheral blood mononuclear cells (PBMCs) in the early stages of RA, in order to develop a novel diagnostic modality.

**Methods and Findings:**

PBMCs were isolated from individuals diagnosed with early RA (ERA) (n = 38), longstanding RA (n = 10), osteoarthritis (OA) (n = 19) and from healthy individuals (n = 10). PBMCs were examined for activation of 15 signaling effectors, using phosphorylation status as a measure of activation in immunophenotyped cells, by flow cytometry (phospho-flow). CD3+CD4+, CD3+CD8+ and CD20+ cells isolated from patients with ERA, RA and OA exhibited activation of multiple phospho-epitopes. ERA patient PBMCs showed a bias towards phosphorylation-activation in the CD4+ and CD20+ compartments compared to OA PBMCs, where phospho-activation was primarily observed in CD8+ cells. The ratio of phospho (p)-AKT/p-p38 was significantly elevated in patients with ERA and may have diagnostic potential. The mean fluorescent intensity (MFI) levels for p-AKT and p-H3 in CD4+, CD8+ and CD20+ T cells correlated directly with physician global assessment scores (MDGA) and DAS (disease activity score). Stratification by medications revealed that patients receiving leflunomide, systemic steroids or anti-TNF therapy had significant reductions in phospho-specific activation compared with patients not receiving these therapies. Correlative trends between medication-associated reductions in the levels of phosphorylation of specific signaling effectors and lower disease activity were observed.

**Conclusions:**

Phospho-flow analysis identified phosphorylation-activation of specific signaling effectors in the PB from patients with ERA. Notably, phosphorylation of these signaling effectors did not distinguish ERA from late RA, suggesting that the activation status of discrete cell populations is already established early in disease. However, when the ratio of MFI values for p-AKT and p-p38 is >1.5, there is a high likelihood of having a diagnosis of RA. Our results suggest that longitudinal sampling of patients undergoing therapy may result in phospho-signatures that are predictive of drug responsiveness.

## Introduction

Rheumatoid arthritis (RA) is a common, relapsing autoimmune disease primarily affecting the joints. RA affects approximately 1% of the population worldwide [1]. The clinical manifestations include joint swelling, deformity, pain, stiffness, and weakness [2]. Within the affected RA joint, there is proliferation of synovial lining cells, pannus accumulation over articular cartilage and erosion of the underlying bone. The rheumatoid synovium is an area of intense immunological activity [3], [4] with a profound infiltration of inflammatory cells, including mononuclear cells and lymphocytes, which occasionally form secondary lymphoid structures [5]. Additionally, RA is not exclusively restricted to the joints and other extra-articular manifestation occur and account for considerable mortality and morbidity [6]. While the specific molecular events that lead to initiation and onset of RA are not known, a systemic activation of the immune system is considered to be a critical component of the disease.

The etiology of RA is unclear, however, many cells types including fibroblast like synovial cells (FLS), B and T lymphocytes, macrophages and neutrophils all contribute to joint inflammation. Both T and B lymphocytes have prominent roles in RA pathology. The genetic association of RA with specific HLA-DR1 underscores the importance of T lymphocytes in RA pathology [7]. Additionally, adoptive transfer of CD4+ T cells from affected animals induces joint inflammation in healthy recipients [8], while blocking T cell activation clearly has beneficial consequences in human RA patients [9]. Recently, a novel IL-17 secreting T cell subset (Th17) has been implicated in RA disease pathogenesis in both human RA and in mouse models of disease [10]. B lymphocytes undoubtedly play a critical role in RA pathology, as autoantibodies are found in the majority of patients [11], [12] and B cell depletion with rituximab results in significant improvement in disease symptoms [13]. Additionally, B cells maintain T cell activation in the RA joint [14] and interactions between T and B cells may represent unique events in autoimmune disease [11]. Taken together, the activation of T and B lymphocytes may be early precipitating events in disease pathology and, as such, may identify useful diagnostic markers of disease initiation and/or progression.

Effective management of RA requires early diagnosis and timely treatment to prevent significant joint destruction and improve patient outcomes. Diagnosis of RA is difficult and is based on specific clinical parameters, radiographic evidence of joint destruction and/or the presence of anti-CCP/RF antibodies [12], [15]. The current criteria for diagnosis of RA have come under scrutiny due to an inability to establish the diagnosis of RA in the early stages of the disease [16]. While considerable progress has been made in identifying predictive criteria for disease progression [17], [18], identification of definitive diagnostic markers with higher sensitivity and specificity are required. Early aggressive therapy with multiple DMARDs including biological agents is highly effective in preventing disease progression [19]. Currently there are no specific and/or rapid tests for monitoring disease progression and/or responsiveness to therapy. Viewed altogether, these deficiencies promoted the examination of patient cells for the identification of early activation markers in RA.

Recent advances in flow cytometry have expanded the number of simultaneous analyses to greater than 13, allowing for the detection of both surface and intracellular factors, thereby enabling identification of specific cell subsets as well as their functional activation in a heterogeneous cell population. Phospho-specific flow cytometry (phospho-flow) permits the quantification of phosphorylation levels of intracellular signaling proteins in individual cells, including rare populations of cells [20], [21], [22]. Phospho-flow is highly quantitative [21], [23] and novel network-based screens of complex populations in disease samples can easily be obtained [20]. Phospho-flow technology has identify mutated signaling pathways in leukemia [24], predicted responsiveness of leukemic patients to chemotherapy [25] and can assess the effectiveness of drug therapy in blocking cellular signaling [26]. Accordingly, we have employed phospho-flow technology to analyze the peripheral blood (PB) of patients with early RA (ERA) and established RA and compared the phospho-flow signatures with those from the PB of patients with osteoarthritis (OA) and healthy individuals. In addition, patients were stratified according to medication status and phospho-specific activation was used to examine responsiveness to specific therapies. This exploratory study examined fifteen phospho-epitopes in 3 cell lineages: CD3+CD4+, CD3+CD8+, and CD20+. The data suggest that analysis of the phospho-activation status of the PB leukocyte population may useful for monitoring disease activity and/or responsiveness to therapy.

## Results

### Signaling profiles in RA joint T cells

**Figure 1:**
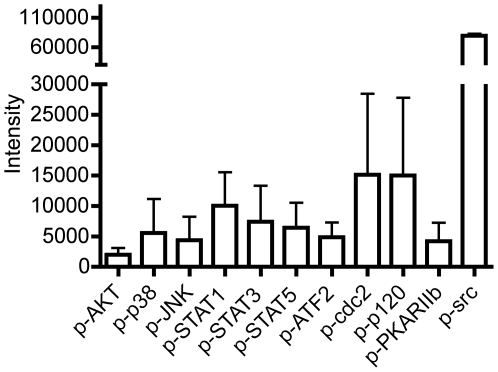
Phospho-specific epitopes are activated in RA synovial tissue lymphocytes. Cell lysates were prepared from ST lymphocytes isolated from the affected joints of 3 patients with late-stage RA, at the time of joint replacement, as described in Methods. Lysates were analyzed by a customized BD PowerBlot and data are shown as mean±SE. Values represent the average intensity of triplicate readings except for p-JNK, p-STAT5, p-cdc2 and p-src, which are averages of duplicate samples.

At the outset, to confirm the activation status of T cells in RA in the context of phosphorylation-activation, we employed a customized BD PowerBlot immunoarray as a high throughput Western blot screen for phosphorylated signaling effectors. 20 paired phospho-signaling effectors were evaluated. CD3+ T cells were isolated from the affected joints of 3 patients with established RA at the time of joint replacement and protein lysates prepared. We identified elevated levels of phosphorylated (p)-AKT, p-p38, p-JNK, p-STAT1, p-STAT3, p-STAT5, p-ATF2, p-cdc2, p-p120, p-PKARIIb and p-src in joint infiltrating CD3+ T cells (Figure 1). Notably, phosphorylation of src is consistent with T cell activation [27].

### Phosphorylation-activation of PB cell subsets in ERA, RA and OA

**Figure 2:**
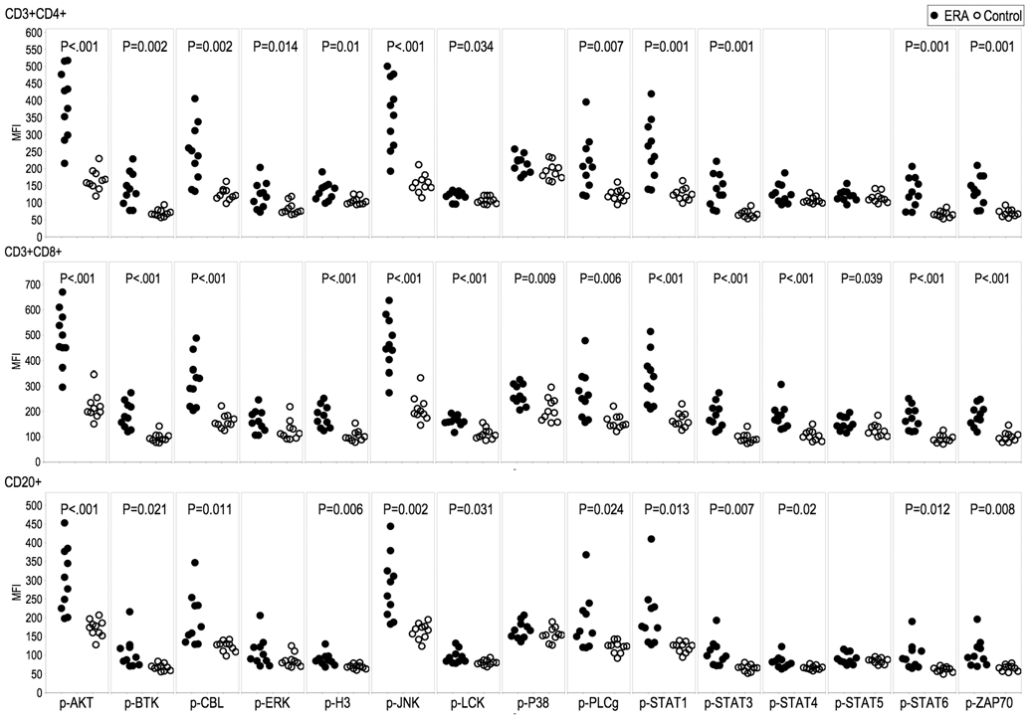
Distinct PBMC subsets in ERA are activated. PBMCs from patients with ERA (n = 10, closed circles) and healthy individuals (n = 10, open circles) were analyzed by multiparameter phospho-FACS, gating on CD3+CD4+, CD3+CD8+ and CD20+ cell populations, as indicated. Scatterplots of the MFI for15 phospho-specific epitopes are shown. Significant differences in MFI values were calculated by Student's t test (p<0.05).

**Figure 3:**
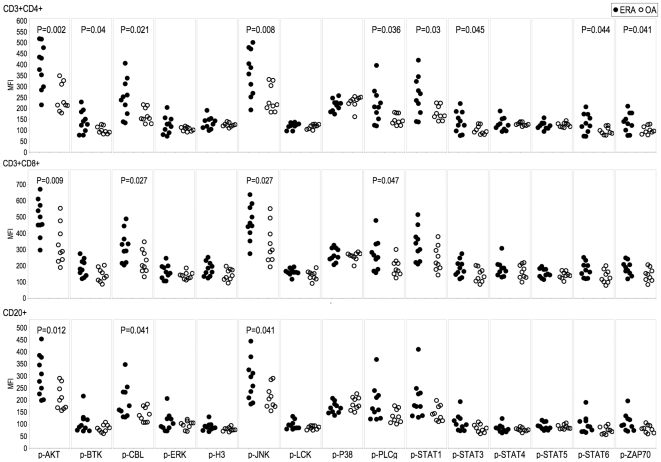
Distinct phosphorylation signatures between ERA and OA PBMCs. PBMCs from patients with ERA (n = 10, closed circles) and OA (n = 9, open circles) were analyzed by multiparameter phospho-FACS, gating on CD3+CD4+, CD3+CD8+ and CD20+ cell populations, as indicated. Scatterplots of the MFI for 15 phospho-specific epitopes are shown. Significant differences were calculated by Student's t test (p<0.05).

Next, multiparameter phospho-FACS was employed to analyze the phosphorylation-activation status of specific signaling effectors in the PB of patients with ERA, established RA and OA, gating on CD3+CD4+, CD3+CD8+ and CD20+ cell populations. The 15 signaling effectors employed in this study were specifically chosen as they are critical signaling nodes activated by multiple pathways and are likely candidates as indicators of activation in multiple cell populations. Multiple phospho-epitopes were activated in the circulating CD4+ and CD8+ T cells and CD20+ B cells from ERA (Figure 2) and established RA patients (data not shown) compared with healthy individuals. Notably, p-AKT, p-CBL, p-JNK, p-PLC-γ, p-STAT1, p-STAT3, p-STAT6 and p-ZAP70 mean fluorescent intensity (MFI) levels were significantly elevated in ERA patient PB subsets in all three cellular compartments. The MFI values were not significantly different between the ERA and established RA patient PB subsets (Figure S1) for any of the 15 phospho-epitopes examined. Because this was an exploratory study investigating the general utility of phospho-flow in diagnosis and treatment, no correction for multiple comparisons was made in Figure 2, Figure S1, or other similar analyses (Please refer to the section on Statistical analysis). If we had corrected using the Bonferroni correction for 45 comparisons (as shown in Figures 2 and 3), a p-value of less than 0.0011 would be considered statistically significant. Rather than discourage the future study of potentially valuable phospho-epitopes by applying such a correction, we instead note that we have not adjusted for family-wise error.

**Figure 4:**
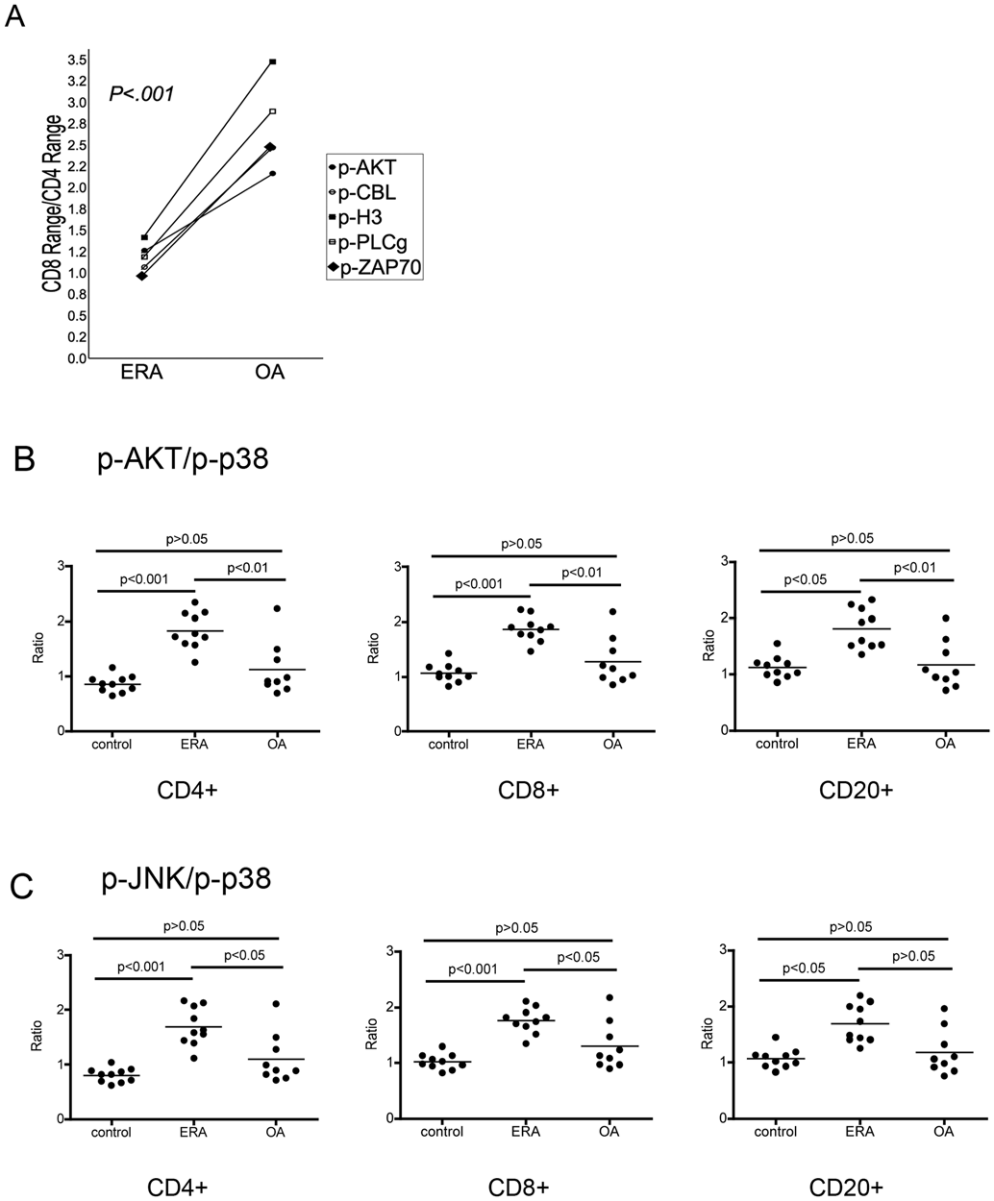
Quantitative differences in phospho MFI values distinguish ERA. The data from Figure 3 are plotted to (A) compare the CD8 MFI range/CD4 MFI range for each of p-AKT, p-CBL, p-H3, p-PLCγ and p-ZAP70 and (B) to compare the p-AKT/p-p38 and p-JNK/p-p38 ratios, between ERA (n = 10) and OA (n = 9) patients, in the indicated cell populations. Significant differences were determined by ANOVA followed by Tukey's multiple comparison (p<0.05).

Next we examined whether phosphorylation of specific signaling effectors was disease specific, by comparing the phosphorylation profiles of PBMC from ERA and OA patients. The MFI levels for p-AKT, p-CBL and p-JNK were statistically higher (p<0.05) in ERA patient PBMCs compared with OA patient PBMCs, in the CD4+, CD8+ and CD20+ compartments (Figure 3). Additionally, the extent of phosphorylation-activation, as measured by the number of phosphorylated signaling effectors for which the MFI values were significantly different between ERA and OA samples, was greatest in the CD4+ T cell population (Figure 3). For each of the phospho-epitopes we next set an arbitrary threshold MFI level that was 10% higher than the maximum MFI level recorded amongst the healthy individuals (Table 1). Our subsequent analysis, based on this threshold, distinguished ERA patients with activated phospho-epitopes in all three cell compartments (CD4+, CD8+ and CD20+) (Table 1). In contrast, OA patients had fewer phospho-epitopes activated, predominantly in the CD8+ T cell compartment (Table 1). Further analysis directly comparing the ERA and OA patient groups, again using a threshold of 10% greater than the highest OA patient MFI value, provided evidence for a distinguishing activation profile in the CD4+ T cells and CD20+ B cells in patients with ERA (Table 2). The data in Figure 4A reveal a significant difference (p<0.001) in the ratio of the CD8 range/CD4 range for p-AKT, p-CBL, p-H3, p-PLCγ and p-ZAP70 between RA and OA patients.

**Table 1:** Elevated phosphorylation of signaling effectors in ERA and OA PB CD4+, CD8+ and CD20+ populations.

Disease	Phospho-signaling effector	CD4+*	CD8+*	CD20+*
ERA	p-AKT	90%	80%	70%
ERA	p-BTK	70%	70%	50%
ERA	p-CBL	70%	70%	60%
ERA	p-H3	50%	50%	40%
ERA	p-JNK	90%	80%	70%
ERA	p-PLCγ	70%	60%	60%
ERA	p-STAT1	80%	70%	70%
ERA	p-STAT3	70%	70%	70%
ERA	p-STAT4	0%	60%	0%
ERA	p-STAT6	70%	70%	60%
ERA	p-ZAP70	70%	70%	70%
OA	p-BTK	0%	44%	0%
OA	p-JNK	0%	0%	44%
OA	p-STAT1	0%	44%	0%
OA	p-STAT3	44%	44%	0%
OA	p-STAT4	0%	44%	0%
OA	p-STAT6	44%	44%	0%

* Percentage of patients with MFI values ≥10% above the maximum level recorded in the healthy individual cohort is denoted.

**Table 2:** ERA PB phospho-signaling is distinguished from OA PB in the CD4+ and CD20+ cell populations.

Phospho-signaling effector	CD4+*	CD8+*	CD20+*
p-AKT	50%	0%	40%
p-BTK	50%	0%	40%
p-CBL	60%	0%	40%
p-JNK	50%	0%	0%
p-PLCγ	60%	0%	40%
p-STAT1	50%	0%	40%
p-STAT3	40%	0%	0%
p-STAT6	40%	0%	40%
p-ZAP70	50%	0%	40%

* Percentage of ERA patients with MFI values ≥10% above the maximum level recorded in the OA patient cohort is denoted.

Scrutiny of ratios of MFI values between pairs of different phosphorylated signaling effectors identified that p-AKT and p-p38 ratios as well as p-JNK and p-p38 ratios distinguished between ERA and OA patients (Figure 4 B, C). Since p-p38 MFI values remained fairly constant across the control, OA and ERA patient specimens, we postulated that p-p38 serves as an internal control to normalize individual samples. Significant differences in the p-AKT/p-p38 ratios were observed in ERA compared with OA in the CD4+, CD8+ and CD20+ cell populations (Figure 4B, p<0.05), whereas the p-JNK/p-p38 ratios were significantly different in the CD4+ and CD8+ populations only (Figure 4C, p<0.05). There were no significant differences in these phospho-pair ratios between the OA and healthy individual cohorts, for any of the three cell types. As anticipated, examination of these phospho-pair ratios in RA patients with established disease, in their PB CD4, CD8 and CD20 cells likewise distinguished RA patients from healthy individuals and OA patients (data not shown). The diagnostic potential of this phospho-epitope ratio was assessed by calculating the percentage of patients with a p-AKT/p-p38 ratio or a p-JNK/p-p38 ratio greater than 1.5. A p-AKT/p-p38 ratio >1.5 was observed in 90% (9/10) of ERA patients and 80% (4/5) of RA patients with established disease, in the CD4, CD8 and CD20 PB cell populations. Notably, this threshold of >1.5 was only exceeded in one of the 10 (10%) healthy individuals in their CD20 cells. For the OA cohort, this ratio was exceeded in one (11%) of the CD4 samples and 2 (22%) of the CD8 and CD20 samples. A p-JNK/p-p38 ratio >1.5 was observed in PB CD4, CD8 and CD20 cells in 70% (7/10), 90% (9/10) and 50% (5/10) of ERA patients, respectively. p-JNK/p-p38 ratio >1.5 was observed in 80% (4/5), 60% (3/5) and 80% (4/5) of RA patients with established disease in their PB CD4, CD8 and CD20 cells, respectively. p-JNK/p-p38 ratio >1.5 was observed in 11% (1/9), 22% (2/9) and 22% (2/9) of OA patients in their PB CD4, CD8 and CD20 cells, respectively. None of the healthy individuals had p-JNK/p-p38 ratios >1.5.

### p-H3 and p-AKT levels correlate with physician global assessment and DAS

**Figure 5:**
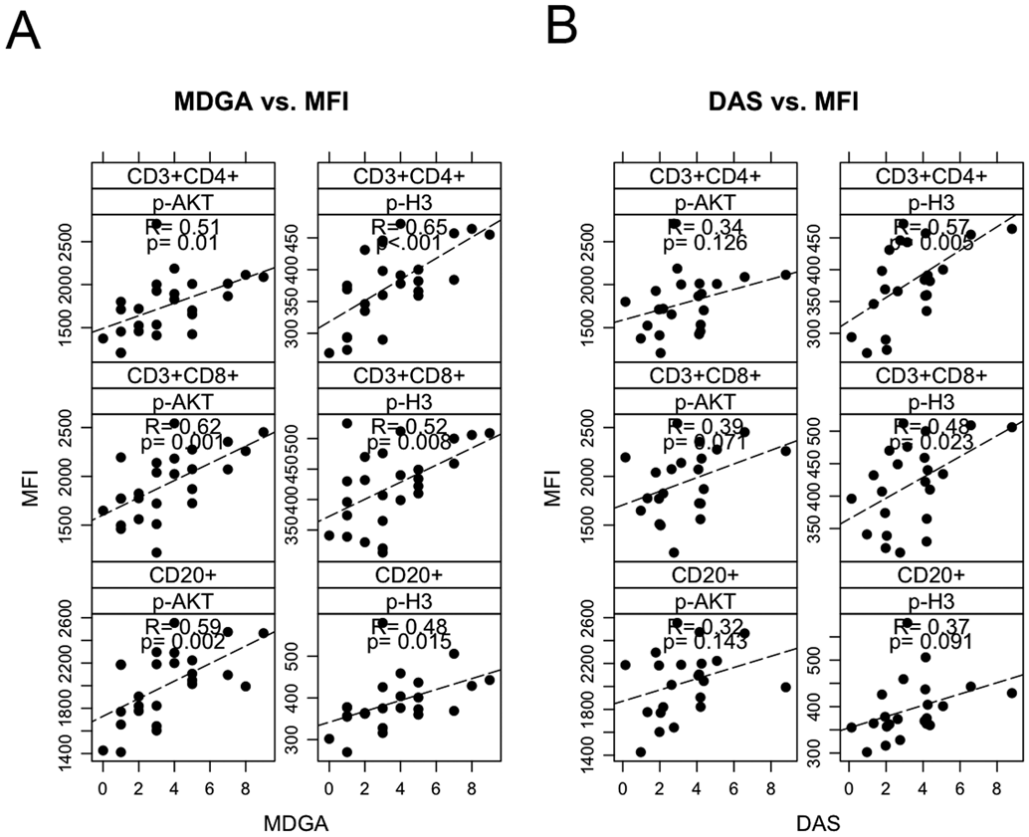
The extent of phosphorylation of H3 and AKT correlates with the MDGA score and DAS28 in ERA PBMC. Dot plots of (A) MDGA vs p-H3 MFI values (n = 32) and (B) MDGA vs p-AKT MFI values (n = 32) in the CD4, CD8 and CD20 cell populations of ERA patient PBMC.

In the next series of analyses, with specimens acquired from a distinct cohort of patients, we examined whether there existed a correlation between phosphorylation status in defined cell populations and disease activity. Clinical parameters measuring disease status (DAS28, physician global assessment (MDGA), CRP and ESR levels) were compared to phospho-activation levels. Correlations were observed between phospho-histone (H3) and p-AKT levels in the CD4+, CD8+ and CD20+ populations in the PB of ERA patients and their MDGA scores (Figure 5). Moreover, the extent of phosphorylation of H3 and AKT in the CD4+,CD8+ and CD20+ cells of the PB of ERA patients also directly correlated with DAS28 (Figure 5).

### Effects of drug therapy on PBMC phospho-activation profile

**Figure 6:**
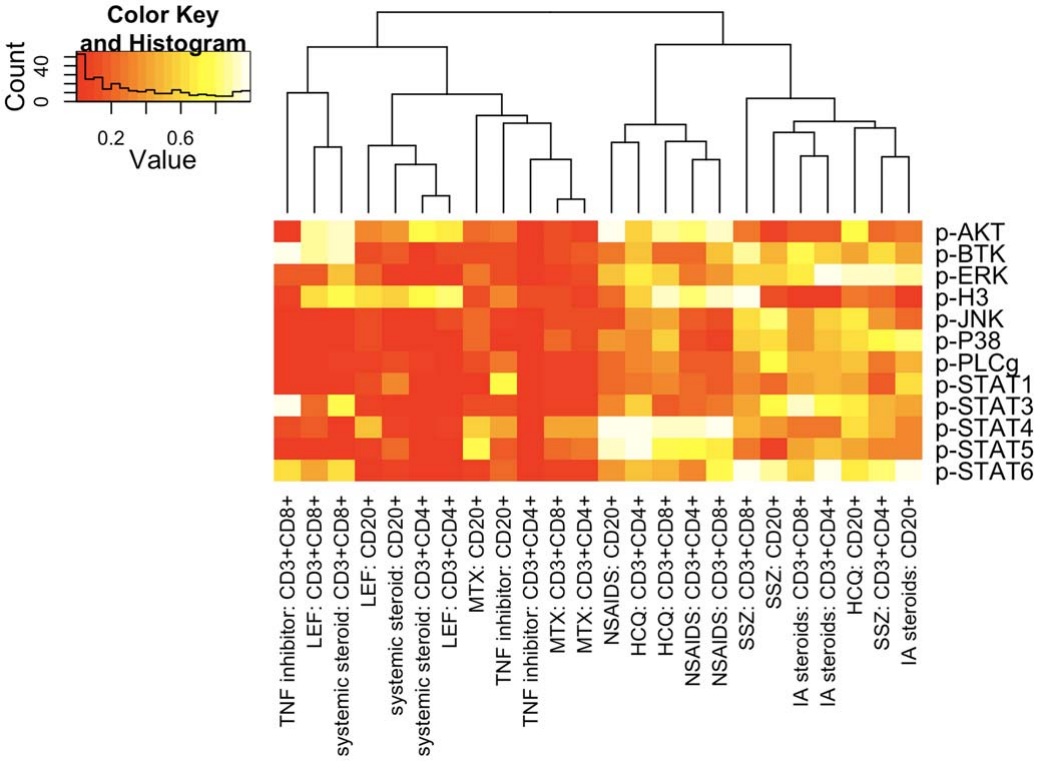
Drug therapy affects the phosphorylation levels of signaling effectors in ERA PBMC. Hierarchical clustering heatmap showing the level of statistical significance, as a p value, for the difference in MFI values for each indicated phospho-signaling effector comparing patients on therapy versus patients not on therapy. Statistical differences were calculated by Student's t test.

We next examined the effects of specific disease modifying anti-rheumatic drugs (DMARDs) on the phosphorylation status of 12 phospho-epitopes in ERA patients. As a first screen, patients were grouped according to whether they were receiving a particular DMARD or not, regardless of whether a patient was on monotherapy or not. All patients in the drug study cohort were on some form of drug therapy (refer to Table S1). The effects of the non-steroidal anti-inflammatory drugs (NSAID), methotrexate (MTX), sulphasalazine (SSZ), plaquenil (HCQ) and leflunomide (LEF), systemic steroids (predominantly prednisone), TNF inhibitors (Enbrel or Humira), and intra-articular (IA) steroids, were assessed. Notably, TNF inhibitors, LEF, systemic steroids and MTX therapy resulted in decreased activation of multiple phospho-eptiopes (Figure 6). Significant differences in MFI values were observed in the PBMC of patients on LEF (Figure 6, Figure S2) or systemic corticosteroids (Figure 6, Figure S3), compared to those patients not taking these specific DMARDs. Notably, 3 of the 5 patients on LEF were also receiving Enbrel. Interestingly, the phospho-activation signatures for each drug were not identical and patients on LEF exhibited reductions in p-STAT6 in their CD20+ B cells and reduced p-PLC-γ in their CD4+ and CD8+ T cells, while patients on systemic steroids showed decreases in p-BTK and p-JNK in their CD4+ cells, p-ERK, p-p38 and p-STAT3 in their CD20+ cells, and p-STAT4 in their CD4+ and CD8+ cells. Patients on TNF inhibitors (Enbrel or Humira) exhibited significant reductions in levels of p-AKT, p-BTK, p-ERK, p-JNK, p-p38, p-PLCγ, pSTAT1, p-STAT3, p-STAT5 and p-STAT6 in their CD4+ cells (Figure 6, Figure S4). Reductions in MFI values for p-AKT, p-p38, p-JNK, p-PLCγ, and p-STAT5 were also observed in their CD8+ cells and reductions in p-p38 were observed in their CD20+ cells.

There were no significant differences in the MFI values between patients prescribed NSAIDS (Figure 6, Figure S5), HCQ (Figure 6, Figure S6) or sulphasalazine (SSZ) (Figure 6, Figure S7) for any of the 12 phospho-epitopes in any of the cell types. Patients receiving IA steroids showed significant reductions in p-H3 in their CD4+, CD8+ and CD20+ cells (Figure 6, Figure S8). MTX therapy reduced p-STAT1 MFI values in CD20+ cells (p = 0.045) (data not shown). Given the effects of LEF and systemic steroid therapy on the activation profile of PBMCs, we re-examined the effects of MTX therapy on phosphorylation status, removing patients on LEF or systemic steroid therapy from these analyses. Our analysis revealed significant reductions in the phosphorylation of AKT and H3 in the PBMC CD4+ cells (Figure S9) but no change in the reduction in p-STAT1 observed in the CD20+ cells. However, reductions in CD20+ cell p-AKT and p-H3 and CD8 cell p-AKT were revealed.

**Figure 7:**
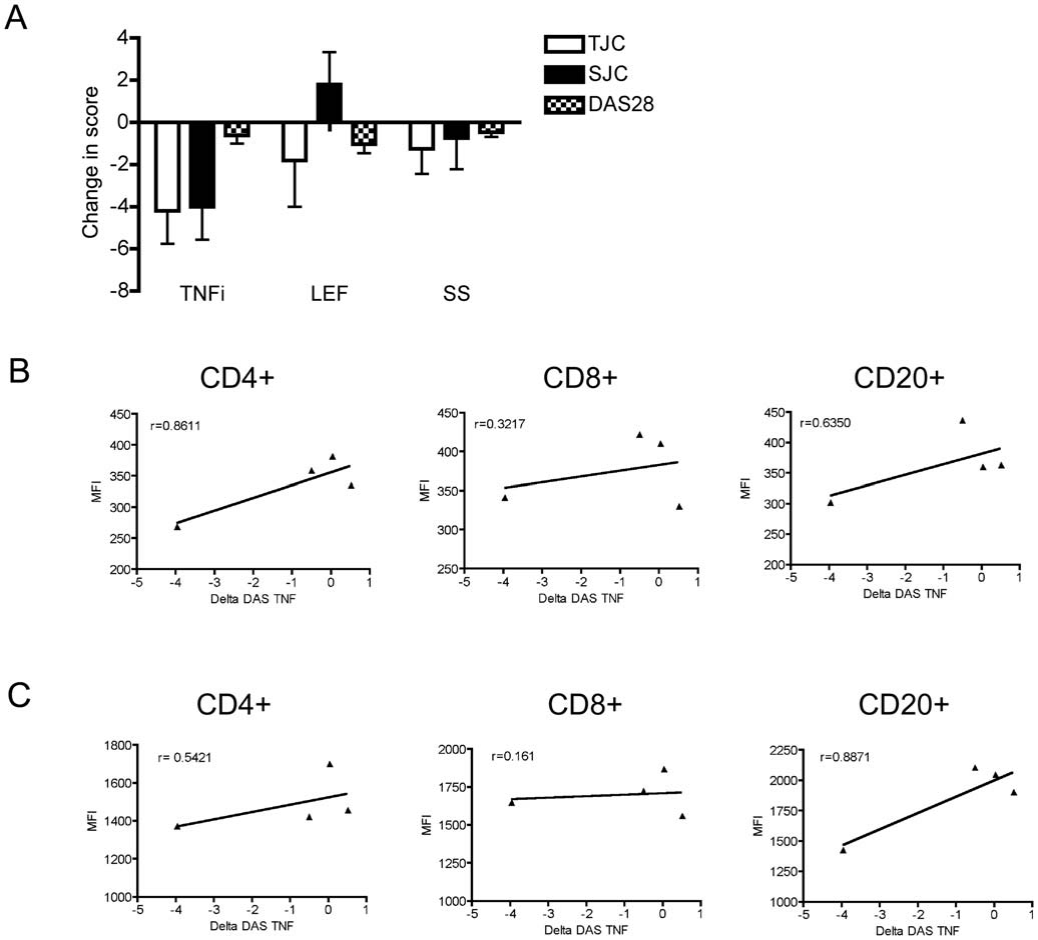
Phosphorylation levels correlate with changes in DAS28. (A) Changes in tender joint count (TJC), swollen joint count (SJC) and DAS28 between the time of sampling and baseline scores are recorded for TNF inhibitors (TNFi) (n = 5, Enbrel or Humira), LEF (n = 6) and systemic steroids (n = 5). Values are the mean±SE. MFI values for (B) p-H3 (n = 4) and (C) p-AKT (n = 4) are plotted relative to changes in DAS in the PB CD4, CD8 and CD20 cell compartments, as indicated.

In a final analysis of these data, we investigated the utility of phosphoflow measurements as correlates of patient responsiveness to therapy. The majority of patients in this study were responding to therapy, as shown by their decreases in DAS (−1.78±0.42, n = 23). Changes in DAS were assessed by subtracting the DAS at the time of sampling with the baseline DAS at their first clinic visit. Of note, some patients were only sampled at their baseline visit and, as such, a delta DAS could not be calculated. Given that anti-TNF therapy affected phospho-signaling effectors to the greatest extent and 3 of the 4 patients on anti-TNF therapy started therapy after their first clinic visit, we selected the data from this cohort for analysis. Patients receiving anti-TNF therapy had an overall decrease in the total tender joint count (TJC), swollen joint count (SJC) and DAS28 (Figure 7A). We observe a high degree of correlation between the DAS and MFI values for p-AKT (Figure 7C) and p-H3 (Figure 7B). These preliminary data suggest that phosphoflow analysis of a limited number of signaling effectors may be reflective of drug responsiveness, at least in the context of anti-TNF therapy, the subject of our ongoing investigations.

## Discussion

RA is a complex, polygenic autoimmune disease that progresses over decades and involves many different cell types, including both resident and recruited cells. Early diagnosis of RA is difficult since there is no single test, but a combination of predictive indices, incorporating clinical parameters, radiographic evidence of joint destruction and the presence of anti-CCP and RF antibodies. The identification of specific activating events that play a role in the pathogenesis and/or progression in RA would be invaluable for both diagnosis and monitoring responsiveness to therapy. Herein, we describe the use of phospho-flow analysis to quantitatively analyze multiple phospho-epitopes as indicators of PB lymphocyte activation.

Lymphocyte infiltration in the RA synovium is observed as either a diffuse inflammatory infiltrate or as clusters of lymphoid follicular aggregates. In some patients, these aggregates exhibit germinal centre-like activity. T cells in affected RA joints show signs of prior activation, are hyporesponsive to antigenic stimulation [28] yet resistant to apoptosis [29], [30]. While we did not compare the level of activation of joint T cells to other inflammatory conditions, our data support previous studies that MAPKs are activated in OA and RA joint tissue [31]. TNF and IL-1, cytokines implicated in disease development, induce activation of these signaling effectors [32]. In addition to p38 and JNK MAPK activation, we observed AKT and STAT activation in CD3+ lymphocytes. AKT promotes cell survival, and MAPKs and STATs are associated with induction of cytokine gene expression, from which we infer that these RA PB lymphocytes may actively participate in the inflammatory response. Whether lymphocyte activation also occurs in the circulation, prior to arrival in the joint, is the subject of these investigations. PB sampling is less invasive than sampling affected joints, and here we show a similar activation profile in ERA PBMCs, from which we infer that lymphocyte activation may precede joint infiltration. While additional validation studies are still required, PB lymphocyte activation may be a reliable indicator/predictor of joint inflammation.

Notably, there were no significant differences in phosphorylation profiles between ERA and established RA PBMCs. The initial stages of autoimmunity involve a breakdown of B and T cell tolerance and in RA this is manifested by measurable levels of serum anti-CCP and/or anti RF antibodies, which are detected years before the onset of articular symptoms [33]. For individuals to have an ERA diagnosis they must satisfy ACR criteria, by which time the mechanisms driving RA are already well established. Since the early RA joint cytokine profile is unique (Th2 skewed) [33], retrospective analysis of inflammatory arthropathies at earlier stages may provide insights into the dynamic changes in PBMCs at the onset of autoimmune disease.

Given the critical roles of CD4+ T cells and CD20+ B cells in autoimmune disease pathology, monitoring dynamic changes in these cell populations may provide insights into disease progression. The 15 signaling effectors employed in this study were specifically chosen as they are critical signaling nodes activated by multiple pathways and as such are likely candidates as indicators of activation in multiple cell populations. Specific signaling effectors (AKT, CBL, JNK, PLC-γ and STAT1) were significantly activated in the PBMC of ERA patients compared with healthy individuals. The MFI values of a subset of these effectors, p-AKT, p-CBL and p-JNK, were consistently higher in all three cell populations in ERA patient PBMCs compared to OA patient PBMCs. Notably, PB CD4+ cells exhibited the largest number of significantly elevated phospho-signaling effectors (Figure 3), underscoring the importance of these cells in autoimmunity. Additionally, a higher percentage of ERA patients showed phospho-activation in their PB CD20+ cells, consistent with B cell hyperreactivity distinguishing RA from OA [11], [12]. However, given the variability across the OA and ERA samples, no single phospho-epitope emerged as a unique diagnostic. Further studies examining the activation status of specific cellular subsets such as CD4+Th17 cells, which are elevated in the circulation of RA patients [34], [35], may be indicative of disease stage and activity. While single phospho-epitopes were not diagnostic, p-AKT:p-p38 ratios and p-JNK:p-p38 ratios >1.5 distinguished between ERA and OA patients (Figure 4B, C). These ratios may be easily adapted into part of a clinical screening regime for the diagnosis of RA.

Our analysis included an examination of correlates between measures of disease activity and severity, or disease status, and levels of phosphorylation of signaling effectors in the different PB cell compartments. We identified correlations between the extent of phosphoryation of AKT and H3 in the CD4+, CD8+ and CD20+ PB cells of ERA patients and their MDGA score and DAS. AKT is a signaling effector associated with cell survival, cell proliferation and cytokine production. Both TNF and IL-17 induce signaling cascades that involve AKT activation. H3 phosphorylation is associated with gene transcription and cell division [36] and the correlation with MDGA score or DAS may point toward expansion of these cell populations.

Currently, factors that are predictive of ‘severity to be’ or responsiveness to particular therapeutic regimens remain, in the main, indicators of likelihood/probability, based on aggregate not individualized data. Many factors have been reported to predict disease progression, responsiveness to therapy or remission, including antibody levels to citrullinated peptides [37], [38] and citrullinated fibrinogen [39], low baseline serum soluble IL-2 receptor levels [40], early response to DMARD treatment [41], [42], the active joint count [43] or urinary levels of C-terminal crosslinking telopeptide of type 1 (CTX-I) and type II (CTX-II) [44]. Generally, RA patients with longer disease duration do not respond as well to treatment compared with patients with early disease, and female sex, prior DMARD use and disease activity [45]. In this context, molecular markers that are accurately predictive in an individual of high risk of rapid progression as well as responsiveness to a particular drug therapy are still required.

Early aggressive treatment of RA is associated with beneficial patient outcomes and there is an accumulating consensus that there is a relatively narrow window of opportunity within which aggressive treatment of RA can produce permanent remission [46]. Early MTX treatment slows disease progression and is the first drug of choice for RA [47], [48]. However, not all patients are responsive to MTX and approximately 50% of patients are refractory to anti-TNF therapy [49]. Current clinical practice involves monitoring therapeutic improvements over several months, whereas phospho-flow analysis represents a rapid alternative. Specifically, medication-induced changes in signature signaling phospho-epitope profiles are rapid. Although in our study cohort the majority of patients were on multiple medications (Table S1), data analysis distinguished the contributions of LEF, corticosteroids, MTX and anti-TNF therapy as signature reductions in phosphorylation profiles.

LEF, MTX and corticosteroids are broad immunosuppressive agents that reduce pain and swelling of affected joints, reduce cytokine production and limit radiological damage [50], [51], [52], [53], [54], [55]. In addition, LEF blocks T and B cell proliferation and activation [56], [57], [58], inhibits TNF dependent NF-κB signaling, reduces matrix metalloproteinase expression and inflammatory cytokine production [51], [59], [60], [61], [62]. Consistent with published data [63], we provide evidence that LEF reduces p-STAT6 levels in CD20+ B cells, which may direct reductions in antibody production. Interestingly, prednisone reduced p-STAT3 levels, and recent data suggest that steroid treatment causes reductions in IL-17 production [64]. Cognizant that STAT3 activation is critical for the development of IL-17 producing T cells [65], [66], it is intriguing to speculate that LEF and prednisone therapy may suppress inflammation via inhibiting the phosphorylation-activation of STAT3. Additionally, LEF inhibits Th17 generation through inhibition of p-STAT6 [67] and we also observed reductions in p-STAT6 levels in patients on these medications. Notably, p-STAT4 levels were dramatically reduced in both the CD4+ and CD8+ T cells in patients on steroid therapy. Certainly, STAT4 has been associated with an increased risk of RA and mice lacking STAT4 are resistant to experimental arthritis [68]. STAT4 expression in joints of patients with ERA has been reported [69] and here we show that prednisone may function in part by blocking STAT4 activation.

Despite its widespread use as a therapy for RA, the mechanisms of action of MTX are complex and not entirely characterized [55]. MTX inhibits dihydrofolate reductase and limits cell proliferation [55]. Therefore, we had anticipated that patients on MTX therapy would exhibit reductions in multiple phospho-signaling effectors, as was the case. Significant reductions in p-AKT and p-H3 MFI values correlated with disease scores. MTX reduces JAK/STAT activation within the joints [70]. Notably, MTX treatment did reduce the levels of phosphorylation of AKT and STAT1.

Results from more than a decade of clinical trials has provided compelling evidence that TNF inhibitors reduce disease activity and delay joint damage [71]. TNF activates multiple kinases (p38, ERK, JNK) and will induce the expression of other pro-inflammatory cytokines that, in turn, invoke pro-inflammatory signaling events associated with kinase activation. Therefore, the substantive therapeutic effects of anti-TNF therapy are a consequence of both direct inhibition of TNF and a blockade of TNF-inducible other pro-inflammatory cytokines. TNF blockade leads to more profound anti-inflammatory outcomes than conventional DMARDs [72] and herein we show that anti-TNF therapy reduced phosphorylation levels of signaling effectors to a greater extent than other DMARDs. Notably the anti-TNF therapy was predominantly initiated during the course of this study, therefore, it is possible that the potency of this drug and sampling close to initiation of therapy gave the most significant effects.

Viewed altogether, these data demonstrate the utility of multi-parameter phospho-flow analysis for monitoring the activation status of discrete PB cell populations in ERA. Quantitative examination of the extent of phosphorylation of critical signaling effectors has the potential to be diagnostic for RA. Moreover, we provide preliminary evidence that reductions in the extent of phosphorylation of these biomarkers may predict responsiveness to drug therapy. Our results suggest that longitudinal sampling of patients undergoing therapy may result in phospho-signatures that not only correlate with drug responsiveness but also may predict patient responsiveness to a specific class of drug. Our ongoing studies are directed to extending these observations in a larger prospective cohort of ERA patients.

## Materials and Methods

### Patients

Sample collection involved confirmation of the diagnosis of RA and OA using clinical, serologic and radiological data. Informed written consent was obtained from all study participants and institutional approval was granted by the Mount Sinai Hospital, St. Michael's Hospital and Sunnybrook and Women's College Health Sciences Centre ethics committees (Toronto, ON). Early RA was defined as patients within the first year following the onset of symptoms with a minimum of 3 swollen joints. Both ERA and established RA patients were diagnosed according to the American College of Rheumatology 1987 revised criteria [15]. The study included a total of 38 ERA patients, 10 late RA patients, 19 OA patients and 10 control, healthy individuals (non-RA, non-OA). The average age of patients in this study was 44.1 years for ERA (range 17–73) and 57.0 years for late RA (range 36–72) with a median disease duration of <1 year for early RA and 10 years for late RA. Clinical parameters were recorded at the time of sample collection and are summarized in Table 3. Multiple samples were collected from some ERA patients and almost all patients in this study were receiving medication (Table S1).

**Table 3:** Patient demographic and clinical characteristics

	Control (n = 10)	ERA (n = 44#)	RA (n = 10)	OA (n = 19)
Age (years)	33.1±8.2	44.1±14.7	57.0±10.7	71.4±9.9
Sex (M/F)	4/6	3/41	1/9	8/11
Anti-RF^+^ pos/neg	ND	4/31	6/2	0/16
Anti-CCP+	ND	ND	9/25	ND
Mean TJC	ND	7.9±7.4 (n = 49)	ND	ND
Mean SJC	ND	6.3±7.1 (n = 49)	ND	ND
Mean HAQ score	ND	0.6±0.65 (n = 48)	1.73±±0.33 (n = 6)	ND
Mean MDGA score	ND	3.8±2.5 (n = 49)	7.60±2.77 (n = 4)	ND
Mean ESR (mm/hr)	ND	17.6±24.1 (n = 46)	30.2±15.5 (n = 6)	16.8±13.4 (n = 14)
CRP (mg/L)	ND	11.9±32.6 (n = 42)	16.9±18.1 (n = 8)	4.3±2. 8 (n = 17)
WBC (×10^9^/L)	ND	7.1±2.3 (n = 46)	7.60±2.76	7.91±2.05
% on corticosteroids	ND	20% (n = 40)	37.5% (n = 8)	0% (n = 11)
% on MTX	ND	45% (n = 40)	66.7 (n = 9)	0% (n = 11)

RF ∼ rheumatoid factor- negative result is <20 IU/ml; CCP ∼ anti-cyclic citrullinated peptide antibody positive; TJC ∼ tender joint count; SJC ∼ swollen joint count; HAQ ∼ Health Assessment Questionnaire; MDGA ∼ physician's global assessment, ESR ∼ erythrocyte sedimentation rate; CRP ∼ C Reactive protein; WBC ∼ white blood cell count; corticosteroids included prednisone and florinef;

# ∼ 5 patients were sampled on 2 separate occasions.

ND ∼ not determined.

* data are shown as mean±SD.

### Synovial tissue

RA synovial tissue (ST) samples (n = 3) were collected from patients with erosive, end-stage RA at the time of joint replacement surgery. All ST specimens were immediately transferred to research personnel for processing.

### Blood collection

Human PB was collected into heparinized vacutainer tubes and PBMCs were isolated by Ficoll gradient centrifugation at 800 g for 20 minutes. The PBMC layer was washed, counted, resuspended in 90% human serum (Irvine Scientific, Irvine, CA) and 10% DMSO (Sigma, St Louis, MO) and frozen in liquid nitrogen to maintain cellular activation levels. Blood samples from control, healthy adults (University Health Network, Toronto) were handled and processed in parallel.

### Cell surface and intracellular phospho-specific flow cytometry

For flow cytometric analysis, human PBMCs were thawed at 37°C, washed twice and incubated for 1 hour. PBMC were then fixed in 2% paraformaldehyde at 37°C for 10 minutes. Cells were counted, pelleted and resuspended at 1×10^7^/ml in FACS buffer. Cells were washed and permeabilized with 90% ice-cold methanol for 15 min: PBMCs were stained with directly conjugated antibodies against CD4 (RPA-T4), CD8 (RPA-T8), CD20 (L27) and phospho-epitopes (Becton-Dickinson; San Jose, CA, unless otherwise specified) as previously described [22](Table S2). Intracellular stains consisted of phospho-specific proteins for p-AKT (Biosource, pS473), p-BTK (pY551), p-PLC-γ (pY783), p-cbl (pY700), p-H3 (Cell Signaling Technology, pSer10), p-JNK (Biosource, pTpY183/185), p-p38 (pT180/Y202), p-p44/42 (pT201/Y202), p-lck (pY505), p-ZAP70 (pY319), p-STAT1 (pY701), p-STAT3 (pY705), p-STAT4 (pY693), p-STAT5 (pS727) and p-STAT6 (pY641). Cells were washed twice and 100,000-500,000 cells were acquired on an LSRII FACS machine operated at Stanford University (12 color, BD Biosciences) acquired with DiVa software (Beckton Dickinson) and analyzed with Flojo (Treestar). Data were acquired on an initial set of samples (controls n = 10, ERA n = 10, RA n = 5, OA n = 9) and a second and larger cohort of samples (ERA n = 40, RA n = 10, OA n = 10) was used to assess correlations and drug related effects.

### Synovial lymphocyte isolation and BD PowerBlot analysis

CD3+ T cells from ST from affected RA joints were isolated by enzymatic digestion with collagenase I, II, IV (3 mg/g tissue) and DNase (2 mg/g tissue) for 0.5–2 hours at 37°C, depending on the sample size. Single cell suspensions were collected after straining the digest through a 70 µm filter. T cells were purified by negative selection, using 0.1″ StemSep magnetic columns and antibodies against CD32, CD19, CD56, CD66b, glycophorin A, 5E11 and dextran. Contaminating macrophages were removed by 2 hour incubation on tissue culture plastic. CD3+ T cell lysates were collected in lysis buffer (10 mM Tris,pH 7.4, 1 mM sodium orthovanadate, 1% SDS), sonicated and frozen at −80°C. Protein samples (200 µg) were resolved by electrophoresis on a 4–15% gradient SDS-polyacrylamide gel and transferred onto an Immobilon-P membrane (Millipore) and analyzed as previously described [73]. Samples were analyzed by a customized phosphoprotein BD array using the average of duplicate (n = 2) or triplicate (n = 1) readings. 57 proteins were assessed in these arrays.

### Statistical analysis

Results are expressed as the mean±SE, unless otherwise indicated. Populations were compared with Student's t-test with no assumption of equal variance, unless otherwise noted. P-values less than 0.05 were considered statistically significant. Correlation coefficients and associated p-values were computed using linear regression. ANOVA was performed on samples with multiple groups followed by Tukey's Multiple Comparison test. Statistical tests were performed using either the R environment for statistical computing (www.r-project.org) or Prism (GraphPad Software, San Diego CA).

## Supporting Information

Figure S1 PBMC activation patterns are similar in RA and ERA. PBMCs from patients with RA (n = 5, open circles) and ERA (n = 10, closed circles) were analyzed by multiparameter phospho-FACS, gating on CD3+CD4+, CD3+CD8+ and CD20+ cell populations, as indicated. Scatterplots of the MFI for 15 phospho-specific epitopes are shown. No significant differences in MFI values were identified, calculated by Student's t test (p<0.05).
(2.37 MB TIF)

Figure S2 LEF affects the phosphorylation status of ERA PBMCs. PB MFI values for each of the indicated phospho-epitopes are plotted to compare ERA patients on LEF (n = 6 except for p-AKT, p-BTK and p-H3 where n = 4; open circles) versus those not receiving this DMARD (n = 31 except for p-JNK, p-p38 and p-STAT3 where n = 30 and p-AKT, p-BTK and p-H3 where n = 19; closed circles). Data are shown for each of the indicated cell populations. Significant differences were calculated by Student's t test (p<0.05).
(1.02 MB TIF)

Figure S3 Systemic steroids affect the phosphorylation status of ERA PBMCs. PB MFI values for each of the indicated phospho-epitopes are plotted to compare ERA patients on systemic steroids (n = 9 except for p-AKT, p-BTK and p-H3 where n = 6; open circles) versus those not (n = 28 except for p-JNK, p-p38 and p-STAT3 where n = 27 and p-AKT, p-BTK and p-H3 where n = 17; closed circles). Data are shown for each of the indicated cell populations. Significant differences were calculated by Student's t test (p<0.05).
(1.12 MB TIF)

Figure S4 TNF inhibitors lower the activation status of ERA PBMCs. PB MFI values for each of the indicated phospho-epitopes are plotted to compare ERA patients on TNF inhibitors (n = 5 except for p-AKT, p-BTK, p-JNK, p-p38, p-H3 and p-STAT3 where n = 4; Enbrel or Humira, open circles) versus those not receiving anti-TNF therapy (n = 32, except for p-AKT, p-BTK and p-H3 where n = 19; closed circles). Data are shown for each of the indicated CD4+ cell populations. Significant differences were calculated by Student's t test (p<0.05).
(1.18 MB TIF)

Figure S5 NSAID therapy does not affect phosphorylation- activation in ERA PBMCs. PB MFI values for each of the indicated phospho-epitopes are plotted to compare ERA patients on NSAIDs (n = 20 except for p-JNK, p-p38 and p-STAT3 where n = 18 and p-AKT, p-BTK and pH3 where n = 12; open circles) versus those not (n = 17 except for p-AKT, p-BTK and p-H3 where n = 11; closed circles). Data are shown for each of the indicated cell populations. NSAIDS included ibuprophen, naprosyn, Arthrotec, Diclonefac, Bextra, Celebrex, Mobicox, Meloxicam and Vioxx. Significant differences were calculated by Student's t test (p<0.05).
(2.43 MB TIF)

Figure S6 The effects of Plaquenil therapy on phospho-signaling in ERA PBMCs. PB MFI values for each of the indicated phospho-epitopes are plotted to compare ERA patients on plaquenil (n = 22 except for p-AKT, p-BTK and p-H3 where n = 14; open circles) versus those not (n = 15 except for p-JNK, p-p38 and p-STAT3 where n = 14 and p-AKT, p-BTK and p-H3 where n = 9; closed circles). Data are shown for each of the indicated cell populations. Significant differences were calculated by Student's t test (p<0.05).
(2.41 MB TIF)

Figure S7 The effects of Sulphasalazine therapy on phospho-signaling in ERA PBMCs. PB MFI values for each of the indicated phospho-epitopes are plotted to compare ERA patients on sulfasalazine (n = 9 except for p-AKT, p-BTK and p-H3 where n = 4; open circles) versus those not (n = 28 except for p-JNK, p-p38 and p-STAT3 where n = 27 and p-AKT, p-BTK and p-H3 where n = 19; closed circles). Data are shown for each of the indicated cell populations. Significant differences were calculated by Student's t test (p<0.05).
(2.32 MB TIF)

Figure S8 The effects of IA steroid therapy on phospho-signaling in ERA PBMCs. PB MFI values for each of the indicated phospho-epitopes are plotted to compare ERA patients on intra-articular (IA) steroids (n = 17 except for p-JNK, p-p38 and p-STAT3 where n = 16 and p-AKT, p-BTK and p-H3 where n = 11; open circles) versus those not (n = 20 except for p-AKT, p-BTK and p-H3 where n = 12; closed circles). IA steroids included DepoMD, Kenalg1 and dexamethasone. Data are shown for each of the indicated cell populations. Significant differences were calculated by Student's t test (p<0.05).
(2.33 MB TIF)

Figure S9 Methotrexate therapy decreases phospho-signaling in ERA PBMCs. PB MFI values for each of the indicated phospho-epitopes are plotted to compare ERA patients on MTX (no LEF or systemic steroids) (n = 15 except for p-JNK, p-p38 and p-STAT3 where n = 13 and p-AKT, p-BTK and p-H3 where n = 8; open circles) versus those not (n = 12 except for p-AKT, p-BTK and p-H3 where n = 9; closed circles). Data are shown for each of the indicated cell populations. Significant differences were calculated by Student's t test (p<0.05).
(2.42 MB TIF)

Table S1 Patient medication
(0.08 MB DOC)

Table S2 Staining panel for phospho-specific profiling of PB leukocyte subsets. Phospho-specific stains are shown in bold.
(0.03 MB DOC)
