# Supplementary figures and images for: Multiparameter Phospho-Flow Analysis of Lymphocytes in Early Rheumatoid Arthritis: Implications for Diagnosis and Monitoring Drug Therapy
Source: PLoS One. 2009 Aug 20;4(8):e6703. doi: 10.1371/journal.pone.0006703 (PMC2724743; doi:10.1371/journal.pone.0006703)

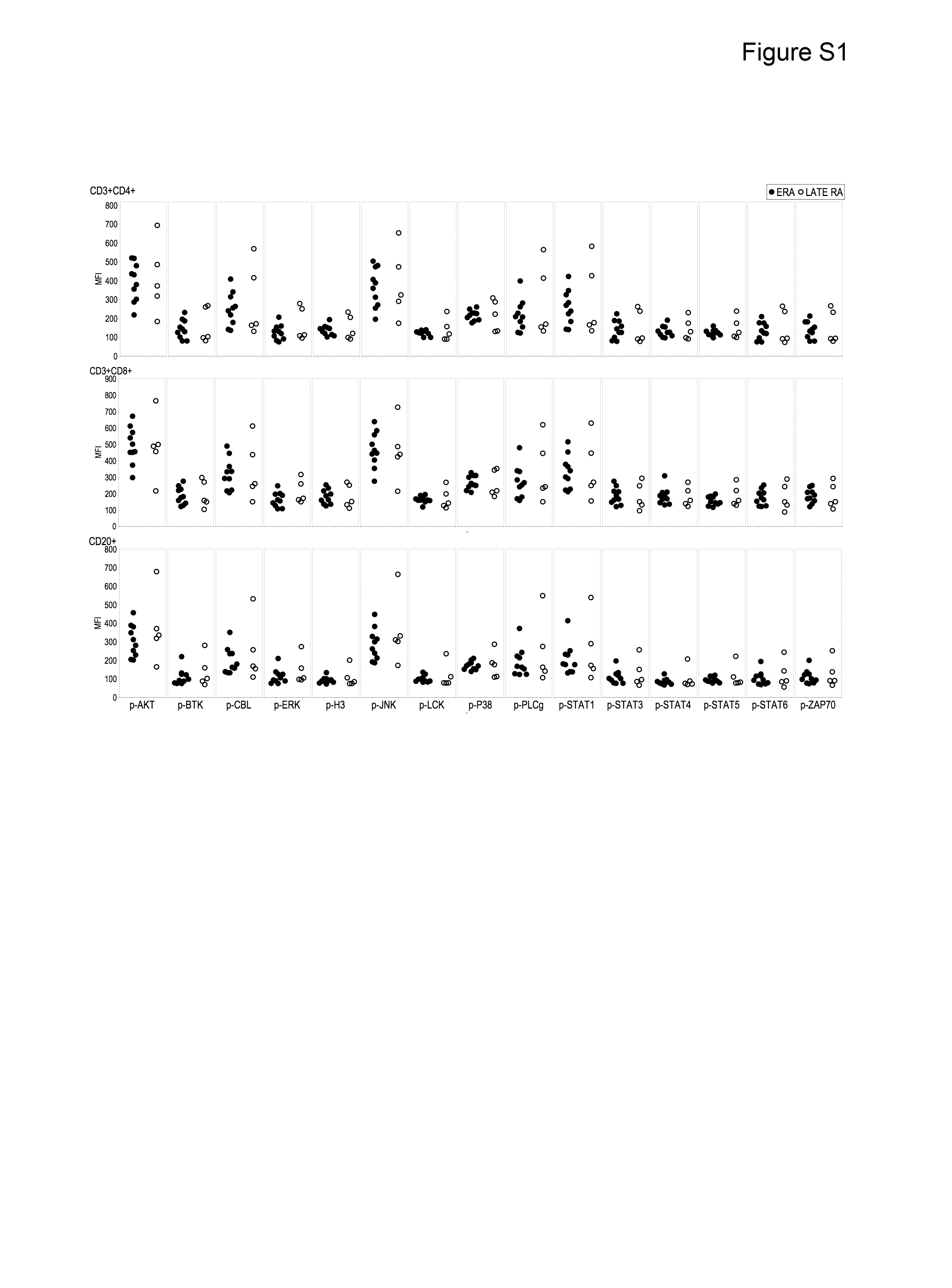

Supplement: Figure S1 — PBMC activation patterns are similar in RA and ERA. PBMCs from patients with RA (n = 5, open circles) and ERA (n = 10, closed circles) were analyzed by multiparameter phospho-FACS, gating on CD3+CD4+, CD3+CD8+ and CD20+ cell populations, as indicated. Scatterplots of the MFI for 15 phospho-specific epitopes are shown. No significant differences in MFI values were identified, calculated by Student's t test (p<0.05). (2.37 MB TIF) [file pone.0006703.s001.tif]

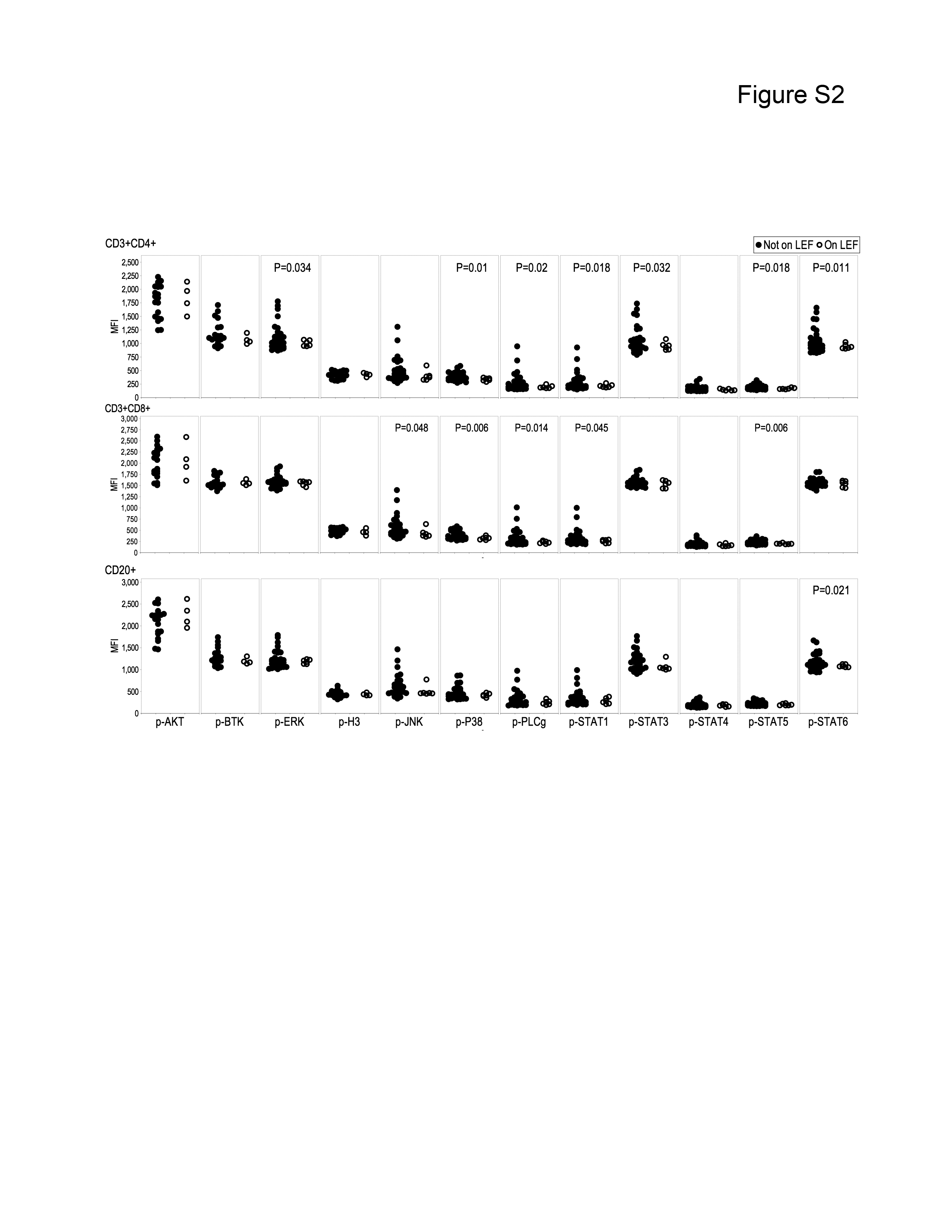

Supplement: Figure S2 — LEF affects the phosphorylation status of ERA PBMCs. PB MFI values for each of the indicated phospho-epitopes are plotted to compare ERA patients on LEF (n = 6 except for p-AKT, p-BTK and p-H3 where n = 4; open circles) versus those not receiving this DMARD (n = 31 except for p-JNK, p-p38 and p-STAT3 where n = 30 and p-AKT, p-BTK and p-H3 where n = 19; closed circles). Data are shown for each of the indicated cell populations. Significant differences were calculated by Student's t test (p<0.05). (1.02 MB TIF) [file pone.0006703.s002.tif]

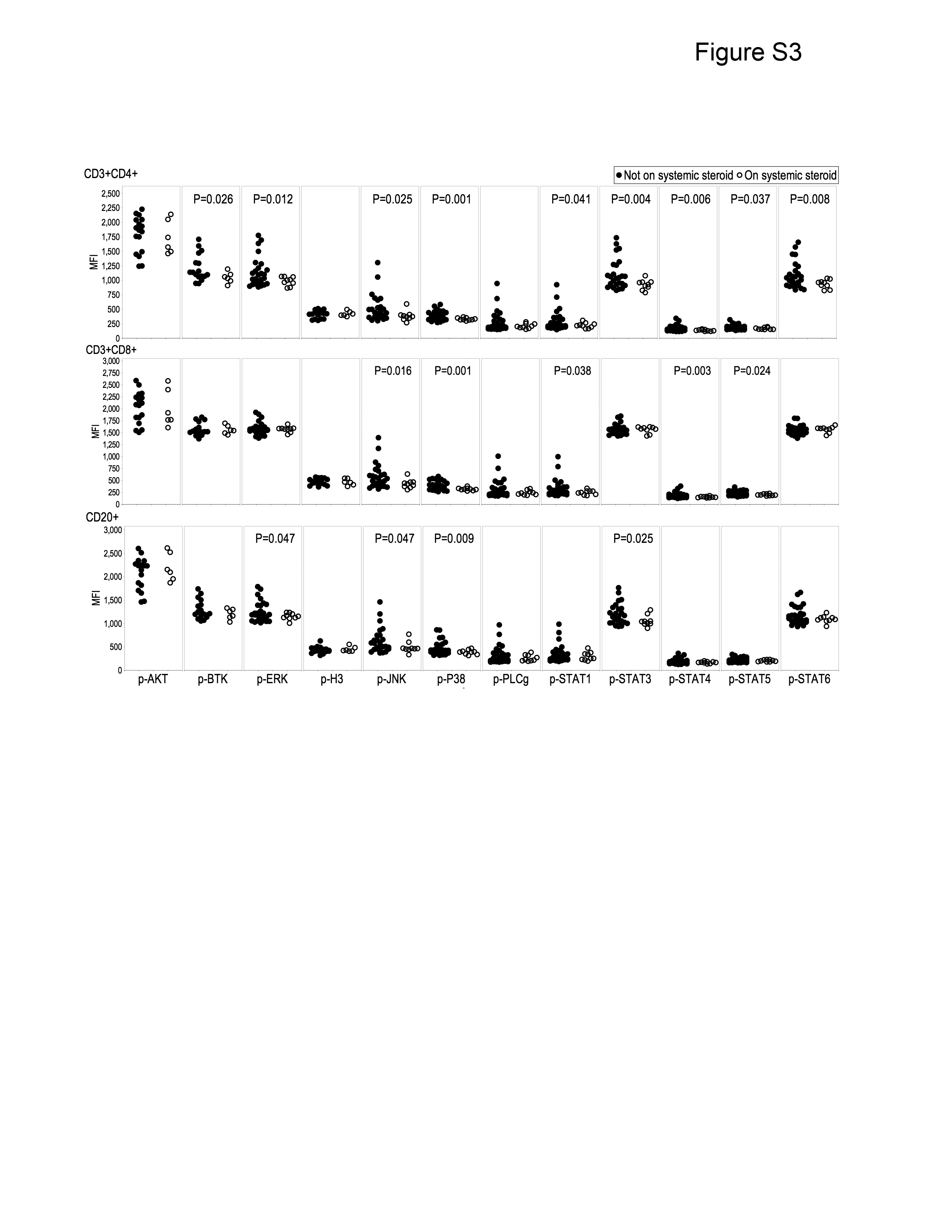

Supplement: Figure S3 — Systemic steroids affect the phosphorylation status of ERA PBMCs. PB MFI values for each of the indicated phospho-epitopes are plotted to compare ERA patients on systemic steroids (n = 9 except for p-AKT, p-BTK and p-H3 where n = 6; open circles) versus those not (n = 28 except for p-JNK, p-p38 and p-STAT3 where n = 27 and p-AKT, p-BTK and p-H3 where n = 17; closed circles). Data are shown for each of the indicated cell populations. Significant differences were calculated by Student's t test (p<0.05). (1.12 MB TIF) [file pone.0006703.s003.tif]

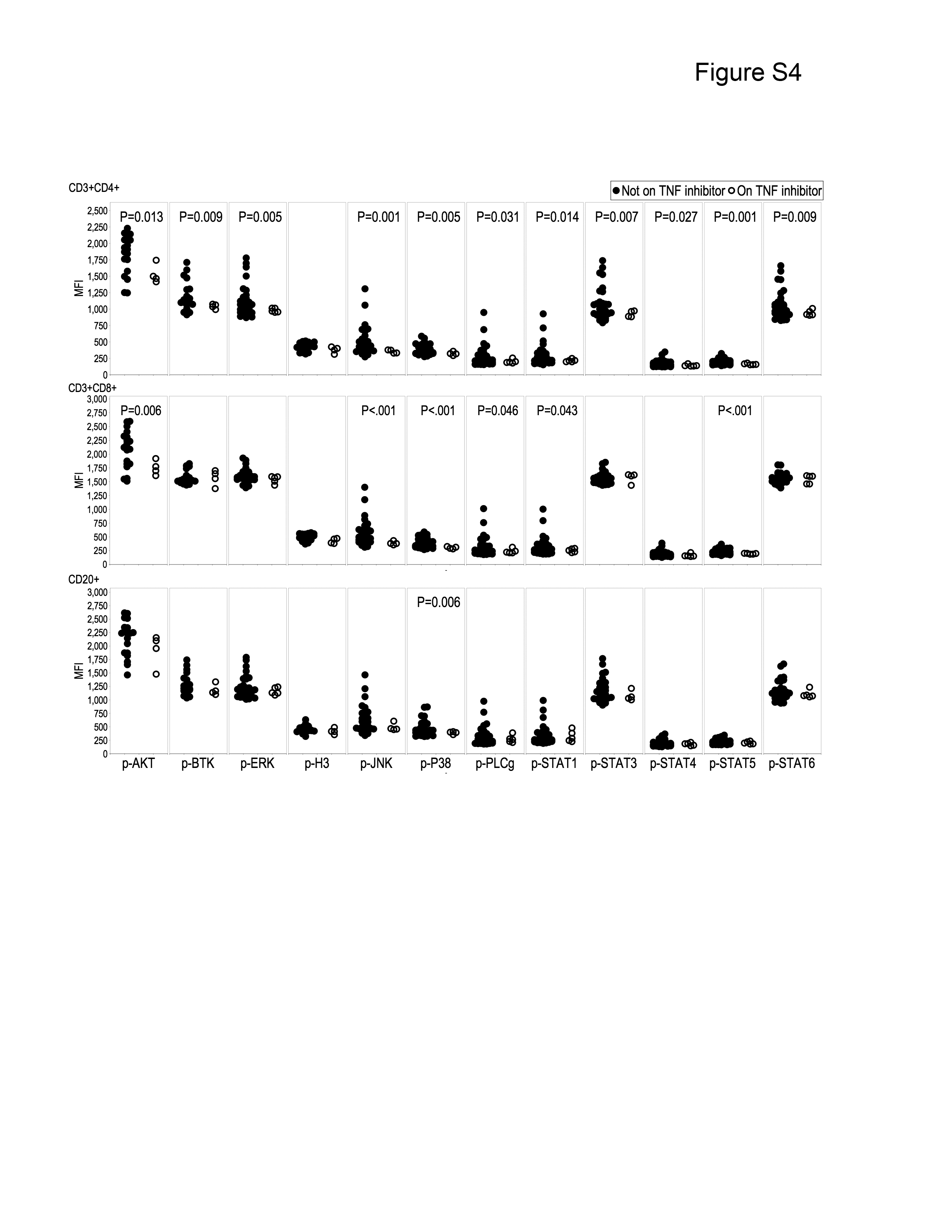

Supplement: Figure S4 — TNF inhibitors lower the activation status of ERA PBMCs. PB MFI values for each of the indicated phospho-epitopes are plotted to compare ERA patients on TNF inhibitors (n = 5 except for p-AKT, p-BTK, p-JNK, p-p38, p-H3 and p-STAT3 where n = 4; Enbrel or Humira, open circles) versus those not receiving anti-TNF therapy (n = 32, except for p-AKT, p-BTK and p-H3 where n = 19; closed circles). Data are shown for each of the indicated CD4+ cell populations. Significant differences were calculated by Student's t test (p<0.05). (1.18 MB TIF) [file pone.0006703.s004.tif]

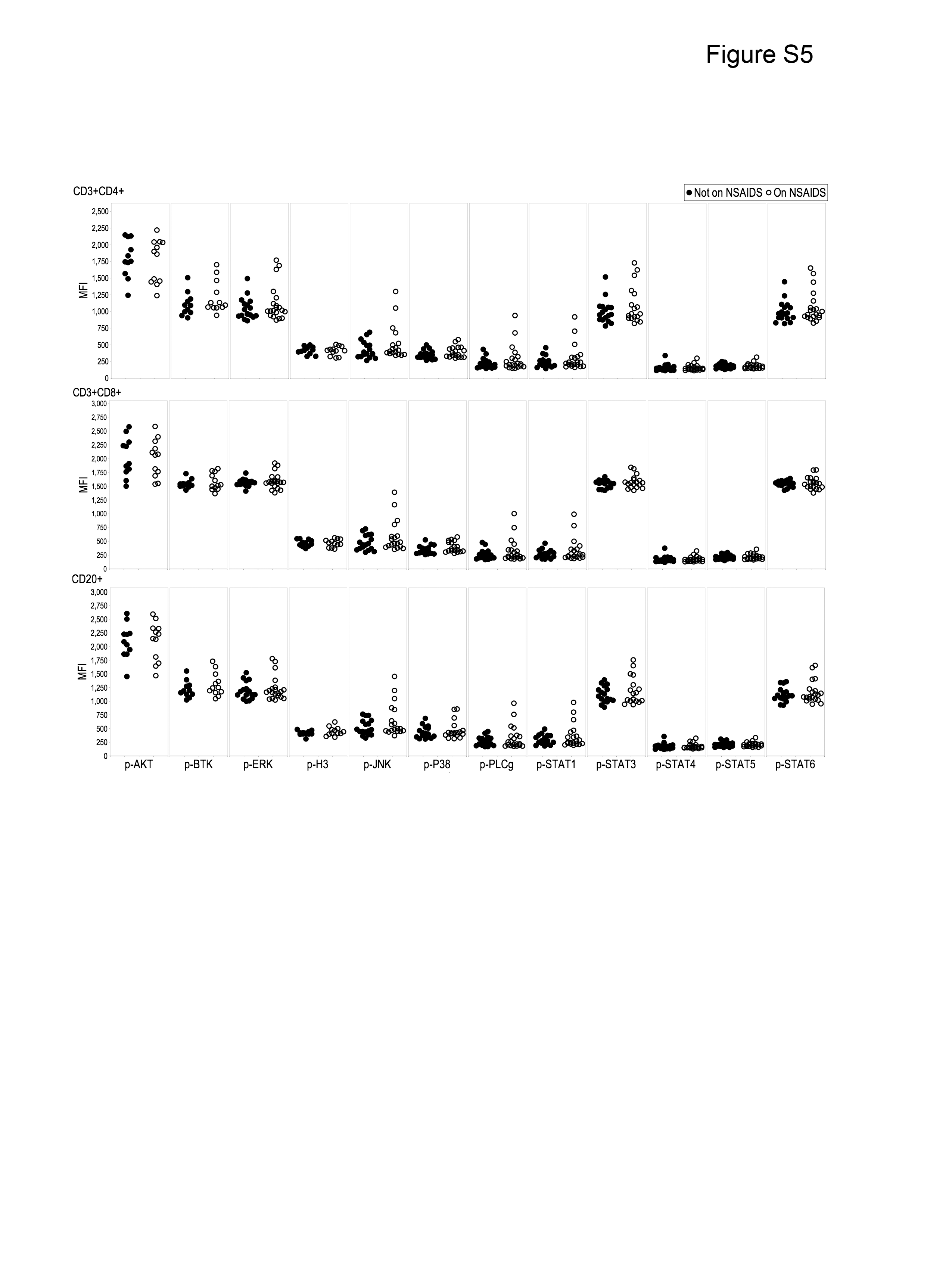

Supplement: Figure S5 — NSAID therapy does not affect phosphorylation- activation in ERA PBMCs. PB MFI values for each of the indicated phospho-epitopes are plotted to compare ERA patients on NSAIDs (n = 20 except for p-JNK, p-p38 and p-STAT3 where n = 18 and p-AKT, p-BTK and pH3 where n = 12; open circles) versus those not (n = 17 except for p-AKT, p-BTK and p-H3 where n = 11; closed circles). Data are shown for each of the indicated cell populations. NSAIDS included ibuprophen, naprosyn, Arthrotec, Diclonefac, Bextra, Celebrex, Mobicox, Meloxicam and Vioxx. Significant differences were calculated by Student's t test (p<0.05). (2.43 MB TIF) [file pone.0006703.s005.tif]

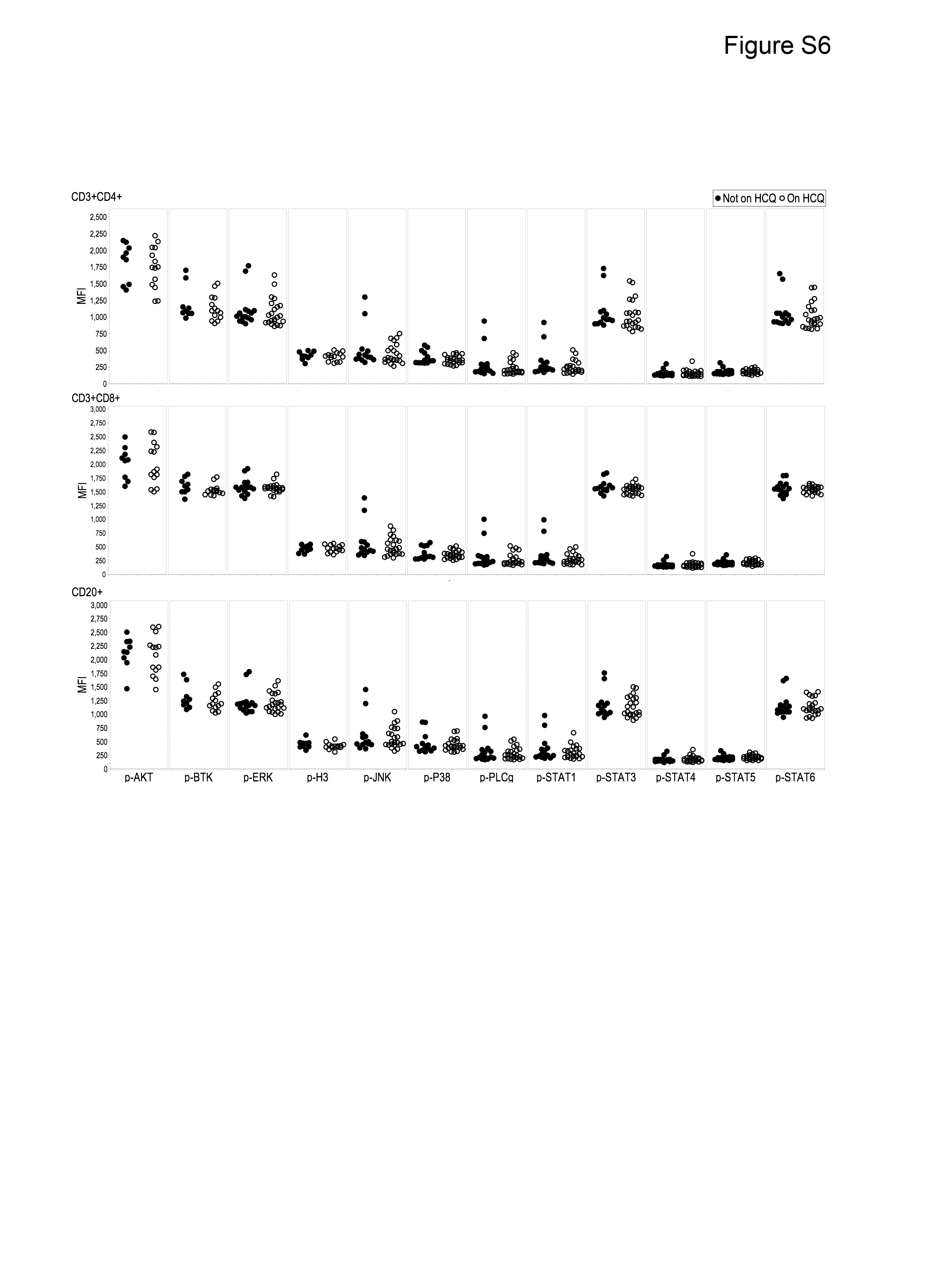

Supplement: Figure S6 — The effects of Plaquenil therapy on phospho-signaling in ERA PBMCs. PB MFI values for each of the indicated phospho-epitopes are plotted to compare ERA patients on plaquenil (n = 22 except for p-AKT, p-BTK and p-H3 where n = 14; open circles) versus those not (n = 15 except for p-JNK, p-p38 and p-STAT3 where n = 14 and p-AKT, p-BTK and p-H3 where n = 9; closed circles). Data are shown for each of the indicated cell populations. Significant differences were calculated by Student's t test (p<0.05). (2.41 MB TIF) [file pone.0006703.s006.tif]

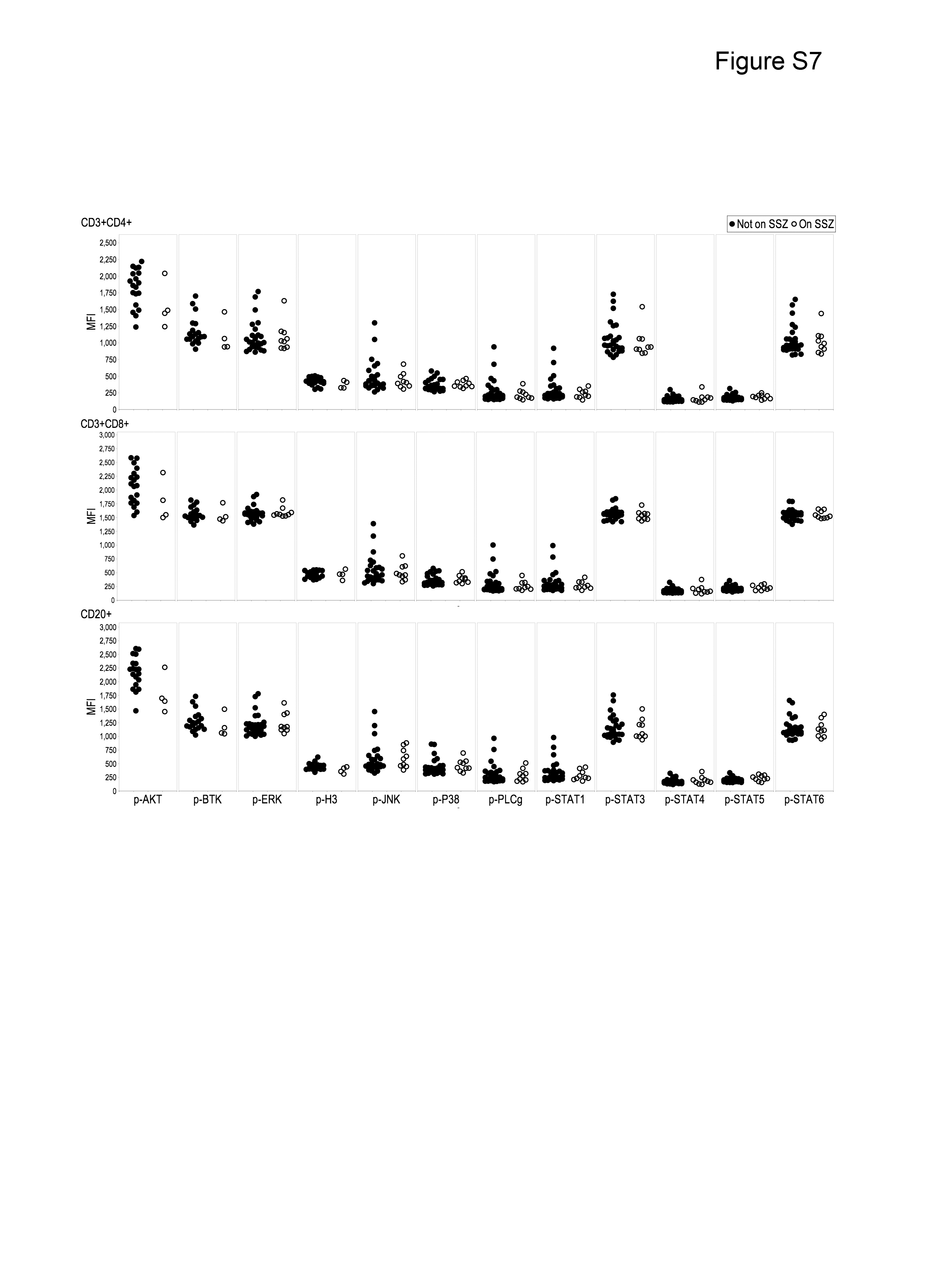

Supplement: Figure S7 — The effects of Sulphasalazine therapy on phospho-signaling in ERA PBMCs. PB MFI values for each of the indicated phospho-epitopes are plotted to compare ERA patients on sulfasalazine (n = 9 except for p-AKT, p-BTK and p-H3 where n = 4; open circles) versus those not (n = 28 except for p-JNK, p-p38 and p-STAT3 where n = 27 and p-AKT, p-BTK and p-H3 where n = 19; closed circles). Data are shown for each of the indicated cell populations. Significant differences were calculated by Student's t test (p<0.05). (2.32 MB TIF) [file pone.0006703.s007.tif]

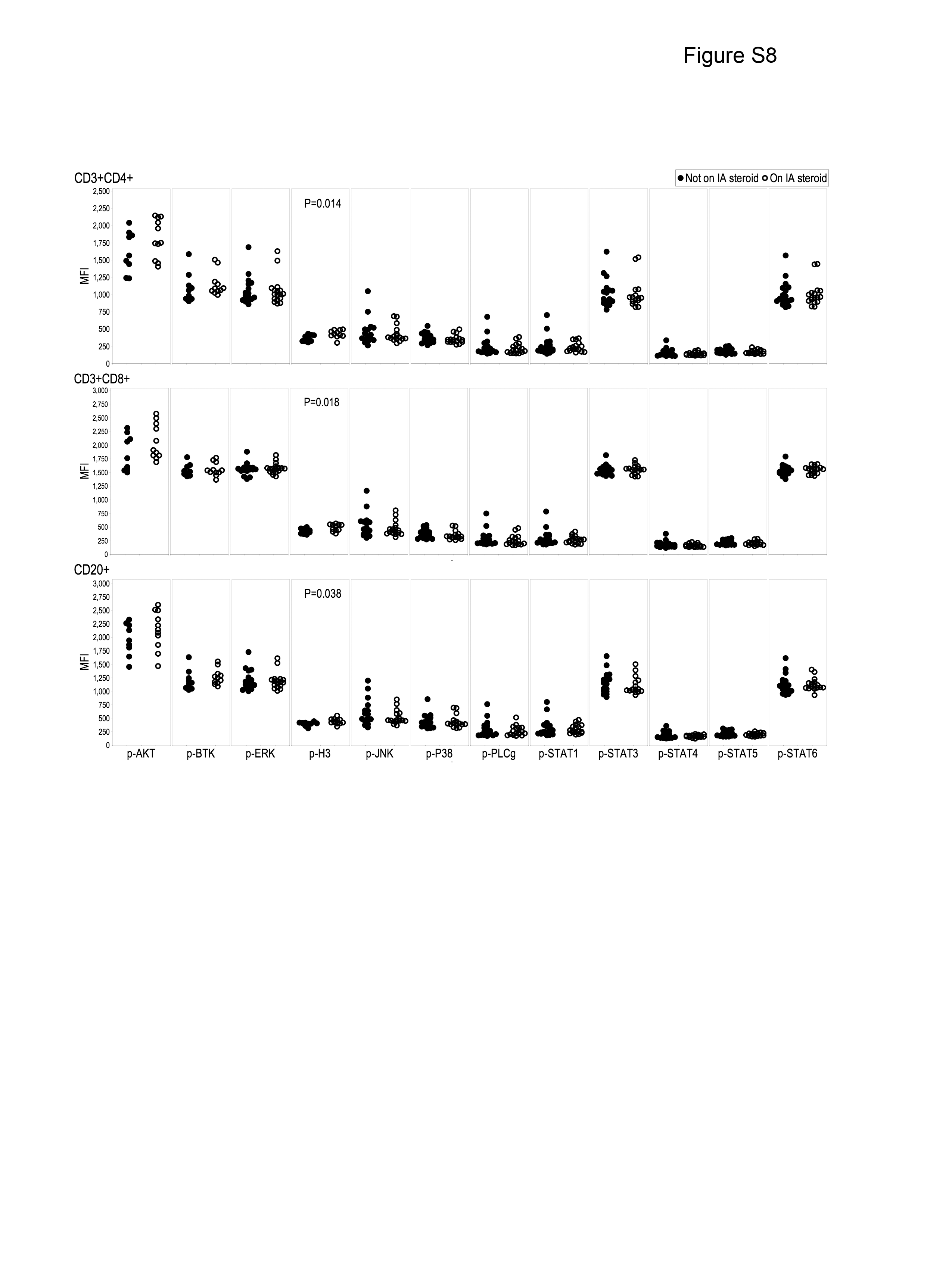

Supplement: Figure S8 — The effects of IA steroid therapy on phospho-signaling in ERA PBMCs. PB MFI values for each of the indicated phospho-epitopes are plotted to compare ERA patients on intra-articular (IA) steroids (n = 17 except for p-JNK, p-p38 and p-STAT3 where n = 16 and p-AKT, p-BTK and p-H3 where n = 11; open circles) versus those not (n = 20 except for p-AKT, p-BTK and p-H3 where n = 12; closed circles). IA steroids included DepoMD, Kenalg1 and dexamethasone. Data are shown for each of the indicated cell populations. Significant differences were calculated by Student's t test (p<0.05). (2.33 MB TIF) [file pone.0006703.s008.tif]

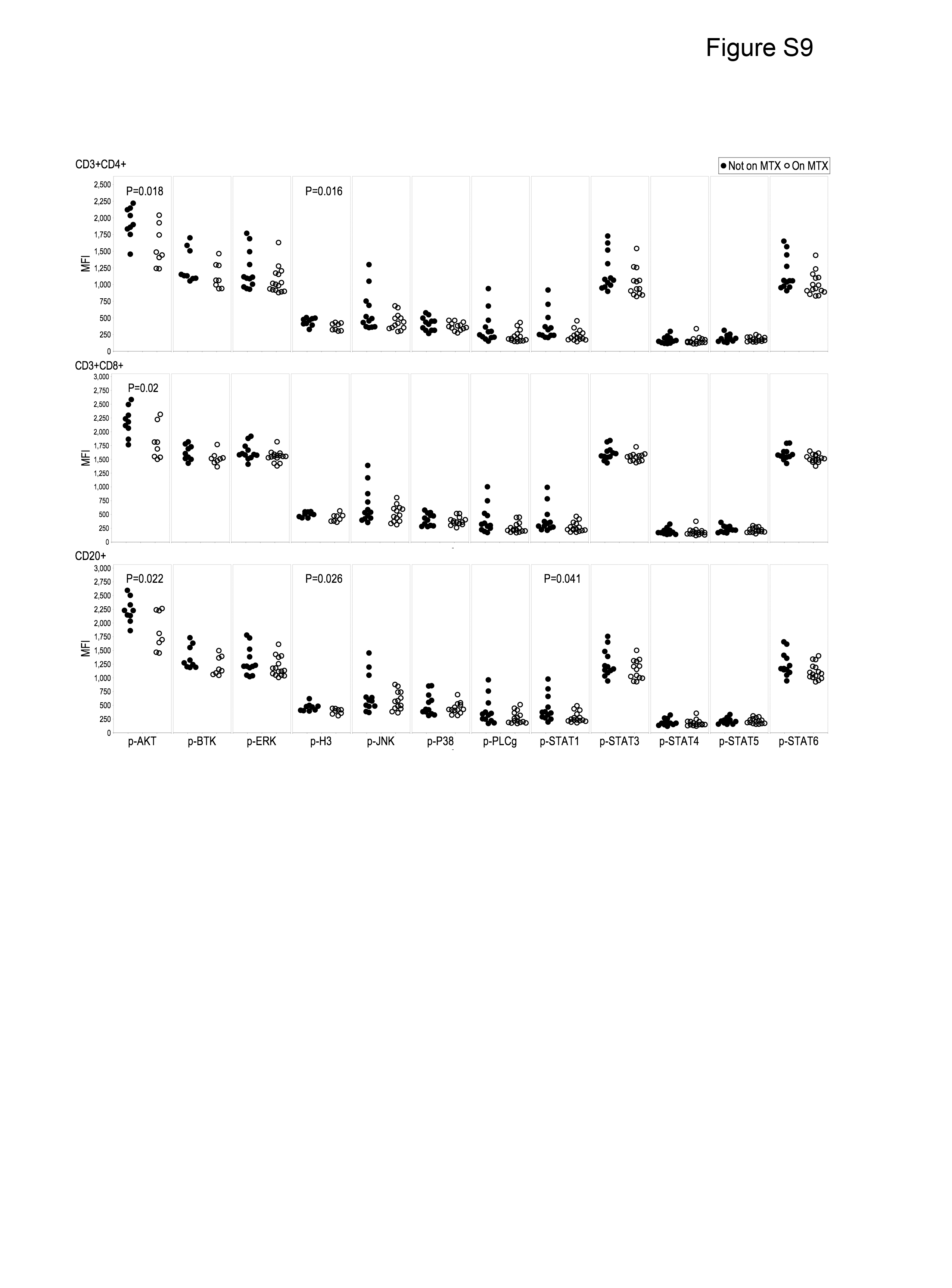

Supplement: Figure S9 — Methotrexate therapy decreases phospho-signaling in ERA PBMCs. PB MFI values for each of the indicated phospho-epitopes are plotted to compare ERA patients on MTX (no LEF or systemic steroids) (n = 15 except for p-JNK, p-p38 and p-STAT3 where n = 13 and p-AKT, p-BTK and p-H3 where n = 8; open circles) versus those not (n = 12 except for p-AKT, p-BTK and p-H3 where n = 9; closed circles). Data are shown for each of the indicated cell populations. Significant differences were calculated by Student's t test (p<0.05). (2.42 MB TIF) [file pone.0006703.s009.tif]
