# Supplementary material for: Multiparameter Phospho-Flow Analysis of Lymphocytes in Early Rheumatoid Arthritis: Implications for Diagnosis and Monitoring Drug Therapy
Source: PLoS One. 2009 Aug 20;4(8):e6703. doi: 10.1371/journal.pone.0006703 (PMC2724743; doi:10.1371/journal.pone.0006703)
Supplement: Table S1 — Patient medication (0.08 MB DOC) [file pone.0006703.s010.doc]

Table S1. Patient medication

| **Patient Identifier** | **Set #** | **NSAIDS** | **MTX** | **SSZ** | **HCQ** | **LEF** | **TNF** | **SS** | **IAS** |
| --- | --- | --- | --- | --- | --- | --- | --- | --- | --- |
| AAE010102073 | 1 | + | + | - | + | - | - | - | - |
| BNE010106359 | 1 | + | - | - | - | - | - | - | - |
| IHE010105876 | 1 | + | + | - | + | - | - | - | - |
| JHE010102243 | 1 | + | - | - | - | - | - | +** | + |
| KME010104268 | 1 | + | + | - | + | + | - | - | + |
| KWE010108147 | 1 | - | - | - | - | - | - | - | - |
| LHE010104076 | 1 | + | + | - | - | + | - | + | + |
| MCE010107578 | 1 | + | - | - | - | - | - | - | - |
| MLE010104357 | 1 | - | + | - | - | + | - | + | - |
| PPE010108648 | 1 | - | - | - | - | - | - | - | - |
| AAE010102073 | 2 | + | + | - | + | - | - | - | - |
| ABE010107731 | 2 | - | + | - | + | + | - | + | + |
| ABE010107731 | 2 | - | + | - | + | + | + | + | + |
| APE010107144 | 2 | + | - | - | + | - | - | - | - |
| ASE010103674 | 2 | - | - | - | + | - | - | - | - |
| ASE010109231 | 2 | + | + | - | + | - | - | - | + |
| CME010100665 | 2 | - | + | + | + | - | - | - | - |
| DJE010110347 | 2 | - | - | - | - | - | - | - | + |
| DOE010109754 | 2 | + | + | + | - | - | - | + | + |
| GZE010110178 | 2 | - | - | - | - | - | - | - | - |
| JDE010104840 | 2 | - | + | - | + | + | +** | - | + |
| JHE010102243 | 2 | + | - | - | - | - | + | +** | + |
| JME010104655 | 2 | + | + | - | - | - | + | - | + |
| JOE010110058 | 2 | - | - | - | - | - | - | - | + |
| JRE010109866 | 2 | - | - | - | + | - | - | + | - |
| JTE010108554 | 2 | + | + | + | + | - | - | - | - |
| JTE010108554 | 2 | + | + | + | + | - | - | - | - |
| KGE010109460 | 2 | - | - | - | - | - | - | - | + |
| KGE010109460 | 2 | - | - | - | + | - | - | - | + |
| KLE010107865 | 2 | - | + | + | + | - | - | - | - |
| KLE010107865 | 2 | - | + | + | + | - | - | - | - |
| KME010104268 | 2 | - | + | - | + | - | - | - | + |
| KWE010108147 | 2 | - | - | + | - | - | - | - | - |
| LBE010100779 | 2 | - | + | + | + | - | - | - | - |
| LHE010104076 | 2 | + | + | - | - | + | - | + | + |
| MBE010109346 | 2 | + | - | - | - | - | - | - | - |
| MKE010109587 | 2 | - | - | - | - | - | - | - | - |
| MLE010104357 | 2 | - | + | - | - | + | + | + | - |
| MRE010105453 | 2 | + | - | - | - | - | - | - | - |
| NPE010103441 | 2 | + | + | - | - | - | - | - | - |
| NPE010103441 | 2 | + | + | - | - | - | - | - | - |
| PME010101244 | 2 | - | + | + | + | - | - | - | - |
| RFE010110476 | 2 | + | - | - | - | - | - | - | - |
| RKE010108247 | 2 | + | - | - | - | - | - | - | - |
| RSE010108468 | 2 | + | + | - | + | - | - | + | + |
| RSE010108468 | 2 | + | + | - | + | - | - | + | + |
| SSE010110265 | 2 | + | - | - | + | - | - | - | - |
| SSE010106777 | 2 | + | + | + | + | - | - | - | + |
| TME010105380 | 2 | + | + | - | - | + | - | - | + |
| VSE010106252 | 2 | - | + | - | + | - | - | - | - |

NSAIDS ~ Non steroidal anti-inflammatory drugs including ibuprophen, naprosyn, Arthrotec, Diclonefac, Bextra, Celebrex, Mobicox, Meloxicam and Vioxx;

MTX ~ methotrexate; SSZ ~ sulphasalazine, HCQBr ~ hydrochloroquine-Plaquinil; LEF ~ Lefluonamide; TNF ~ TNF inhibitor * patient on Humira and all others were on Enbrel; SS ~ systemic steroid * patient was taking florinef and all others were on prednisone; IAS ~ intra-articular steroids
