# Supplementary material for: Multiparameter Phospho-Flow Analysis of Lymphocytes in Early Rheumatoid Arthritis: Implications for Diagnosis and Monitoring Drug Therapy
Source: PLoS One. 2009 Aug 20;4(8):e6703. doi: 10.1371/journal.pone.0006703 (PMC2724743; doi:10.1371/journal.pone.0006703)
Supplement: Table S2 — Staining panel for phospho-specific profiling of PB leukocyte subsets. Phospho-specific stains are shown in bold. (0.03 MB DOC) [file pone.0006703.s011.doc]

Table S2. Staining panel for phospho-specific profiling of PB leukocyte subsets. Phospho-specific stains are shown in bold.

| Stain 1 | Stain 2 | Stain 3 | Stain 4 | Stain 5 |
| --- | --- | --- | --- | --- |
| CD3-cascade blue | CD3-cascade blue | CD3-cascade blue | CD3-cascade blue | CD3-cascade blue |
| **p-PLCγ-Ax488** | **p-Stat1-Ax488** | **p-cbl-Ax488** | **p-jnk-Ax488** | **p-akt-Ax488** |
| **p-p44/42-PE** | **p-Stat6-PE** | **p-Zap70-PE** | **p-Stat3-PE** | **p-BTK-PE** |
| CD20-PERCPCY5.5 | CD20-PERCPCY5.5 | CD20-PERCPCY5.5 | CD20-PERCPCY5.5 | CD20-PERCPCY5.5 |
| CD8-PE-CY7 | CD8-PE-CY7 | CD8-PE-CY7 | CD8-PE-CY7 | CD8-PE-CY7 |
| **p-Stat5-Ax647** | **p-Stat4-Ax647** | **p-lck-Ax647** | **p-p38-Ax647** | **p-histone-H3-Ax647** |
| CD4-APC-CY7 | CD4-APC-CY7 | CD4-APC-CY7 | CD4-APC-CY7 | CD4-APC-CY7 |
